# Hospital and Institutional News

**Published:** 1920-01-03

**Authors:** 


### HOSPITAL AND INSTITUTIONAL NEWS.

### THE DEATH OF SIR WILLIAM OSLER.

The death of the Regius Professor of Medicine at Oxford has removed one of the most notable physicians and "hospital men" in the English-speaking world. It was only a very few months ago that the homage of his own profession was paid him on his seventieth birthday in a well-conceived presentation at the Royal Society of Medicine; and few can then have suspected that his span of useful activity for his fellow-men was drawing so near to its close. Professor, first of all, at the early age of twenty-five in McGill University, Montreal, he moved from there to Philadelphia in the same capacity; but it was his next professorship—at the then newly-constituted Johns Hopkins Hospital and University, Baltimore—that set the seal on his reputation both as teacher and physician. In 1905 he came to Oxford to succeed Burdon Sanderson, and from that time onwards British medicine has been the richer by a ripe and experienced physician, a profound scholar, and pre-eminent teacher—while the Radcliffe Infirmary owes more to him than can easily be expressed. His own researches, though many and valuable, have not put him quite alongside, let us say, a Lister or a James Mackenzie; but in his power of stimulating his students to fruitful research, of encouraging and guiding them, Osler has perhaps been unequalled, or at least unexcelled in our time. His interest in and knowledge of medical history was such as alone to have distinguished him from the ordinary run of consultants; and another of his outstanding qualities was his humanity and his breadth of view. In him neither the heart ruled the head, nor the head the heart. A just balance and a sterling sanity were things he seemed to achieve without effort, because they were the natural foundations of his personality.

### DEATH OF MR F. W HOWELL.

We much regret to record the death of Mr. Fred W. Howell, the well-known Secretary of the Cancer Hospital, which occurred on December 21. Mr. Howell was taken ill suddenly on December 18, and was operated upon the next day for a perforated gastric ulcer at his own hospital. Unfortunately by the time he reached the hospital and was operated on, the ulcer had been perforated for nearly twenty-four hours; and the operation failed to save his life. His funeral took place on Christmas Eve, the first part of the service being held at St. John's Church, West Ealing, previous to the interment at St. George's Cemetery, Hanwell, and a large number of friends, including many associated with Mr. Howell in his work, attended. Mr. F. W. Howell had been secretary at the hospital in the Fulham Road for a great many years. He commenced his institutional career at the Dreadnought Hospital, Greenwich, holding the office of steward. Subsequently he became Secretary to York County Hospital, leaving there to return to the post in London which he held until he died, as no doubt he would wish to die, in harness. Mr. Howell was a past President of the Hospital Officers' Association, and an Honorary Secretary of the Hospital Officers' Club. He leaves a widow, one girl, and one boy, who is at Westminster. To his widow and children the deep sympathy of the whole of the hospital world is accorded.

### THE RESTING-PLACE OF THE FOUNDER OF BART'S HOSPITAL.

An opportunity has arisen to restore a former part of the beautiful church to St. Bartholomew-the-Great, Smithfield, where lies Rahere, the founder of St. Bartholomew's Hospital, in his superb tomb. The church, with its cloisters, is one of the finest in London, and has fortunately escaped, in the intervening centuries since it was built by Rahere, the ruthless hand of the restorer. It still bears some semblance of the days when he walked through it; indeed, few alterations have been made in the structure as it now stands. But many parts have been converted to other uses, including six bays of the east walk, which have been used for many years as a stable. An opportunity has now arisen to purchase these bays, which contain an arched entrance doorway to the chapter house, with the usual window openings on each side; the arched entrance to the dorter, or dormitory, of the monastery, the east side of which was uncovered in 1913; and the entrance to the slype, or passage, between the south transept and the chapter house. There still remains to be raised £1,300 ere the purchase can be completed, and an appeal is being made to those interested in this earliest of tombs of a hospital founder in this country to help to realise the hopes of the rector and those associated with him in restoring yet a little more to this church its ancient precincts.

### AN APPRECIATION.

The Minister of Pensions, Sir Laming Worthington Evans, sent the following Christmas greeting to members of Local War Pensions Committees and members of hospital and other committees working for the Ministry of Pensions:—The past year has been a strenuous one for all those connected with the administration of pensions. The work has been no less difficult and exacting than formerly. I should like to take the opportunity of expressing my appreciation of the splendid way in which all those who, in one capacity or another, working of their own free will and without thought of gain, have helped the nation to pay its great debt to those who suffered and still suffer wounds and sickness through their service in the war. Without the aid of the voluntary workers on the committees it would have been impossible efficiently and sympathetically to carry on the work. To those thus helping I send all good wishes for a very happy Christmas and for the year that is coming.

### HEALTH CONGRESS IN BRUSSELS

The Royal Institute of Public Health has accepted an invitation from the Burgomaster of Brussels (Monsieur Adolphe Max), on behalf of the municipalities of Belgium and the Rectors of the Belgian Universities, to hold their next congress in Brussels, from May 20 to May 24, 1920, inclusive. A letter has been addressed to all local authorities, inviting them to appoint delegates to the congress, and it is urged that the names should be sent in as early as possible, so that full information respecting the meeting, travelling, and hotel accommodation may be sent to them. The fee for each delegate is one guinea, which fee includes all the privileges of the congress. The local authorities should remember that the greatest benefit to public health work has resulted from all former meetings of the Institute, the most notable example being the actual formation of a Ministry of Health, the demand for which emanated at the congress in Dublin in 1892, and was endorsed at each subsequent annual meeting. In this case the advantages offered of acquiring a knowledge of the public health administration of a country other than our own are too obvious to require recounting here.

### THE ELECTRO-MEDICAL DIPLOMA.

During the last few years not only has there been a steady advance in the application of *x*-rays in fracture and localisation work, and in the medical treatment of disease, but parallel with the requirements of this improvement there has also been a marked general complication in the electrical apparatus used. So great is the technical electrical knowledge now required that really efficient work of this type is well-nigh impossible to the average medical man without special training. There have been several agitations made on this point already, and it is good news to read that real efforts are now being made to provide facilities for obtaining this knowledge. Study must be made of the fundamental principles of electro-medical work, and also the technicalities of modern apparatus, before a doctor is in any sense justified in taking it up. A diploma in what is termed " medical radiology and electrology " is to be granted to qualified medical men who fulfil the required conditions and attend a course of theoretical or practical study at the Universities of Cambridge or London.

### ADMINISTRATION OF ANÆSTHETICS.

We are asked to publish some further details in connection with a subject upon which Dr. Waldo, the City Coroner, has lately given much advice. At the request of the jury on a man who died under the influence of an anæsthetic, Dr. Waldo forwarded their unanimous rider to the Home Secretary and Minister of Health, suggesting those recommendations already published in these columns, and also that Dr. Addison and the Medical Research Committee should be requested to inquire into the whole subject of anæsthetics. The Home Secretary replied, stating that the matter was one for Dr. Addison, and that the letter had been forwarded to him. Also in Parliament, Mr. Gilbert (M.P. for Southwark) asked the Home Secretary whether his attention had been drawn to some recent inquests held in London, when it was proved that anæsthetics had been administered in hospitals by unqualified persons, and whether he proposed to introduce legislation to prevent this practice in the future. In course of reply, Dr. Addison stated that he was indeed aware of the matter referred to, and that the subject was receiving his attention in relation to the recommendations of the Departmental Committee on Coroners. With regard to the second paragraph of the question, he was at the time unable to make any statement. So far, so good, and we may commend the determination with which Dr. Waldo is attempting to carry the matter through. At the same time, we trust that he will not find it necessary, in the event of further evidence being collected, specifically to locate his criticisms.

### AN UNUSUAL CASE.

At a recent inquest the house surgeon of the Evelina Hospital explained that the child had been admitted for injuries resulting from an accident, but no fracture of the external plate of the skull could be detected. Later, however, severe symptoms supervening, including a rising temperature, fracture of the internal plate, with cerebral compression, was suspected. X-ray examination showed what appeared to be a fracture of the internal plate, but trephining failed to confirm this. At the *post-mortem* the skull was found to be uninjured and the brain normal, but fracture had occurred of the unossified portion of the epiphysis of the odontoid process of the second cervical vertebra, with resulting pressure on the spinal cord, thus causing the child's death. The father of the deceased child expresses himself as satisfied with the great care and attention which had been given by all those in the hospital to this extremely difficult case.

### ARSENIC AND SYPHILIS.

In view of the surprising lack of published records of the value of intravenous arsenic in the actual cure of syphilitic infections, the recently-presented report by Fildes and Parnell from Haslar Hospital is well worthy of notice. The main conclusions are that: (1) In no early case of syphilis in the stage before the Wassermann test becomes operative was the course of arsenical treatment without mercury known to fail. Failure did occur, however, in certain cases where the Wassermann test was already positive before the treatment began. (2) There was no demonstrable advantage in the use of mercury after the course of arsenical treatment. (3) An early involvement of the central nervous system much increases the liability to relapse. In these conclusions, if they be confirmed by other observers, lies the answer to questions repeatedly asked both by patients and the practitioners of the country. The finer details of their meaning we leave for the reader's own deduction, and need here merely emphasise the paramount importance of immediate diagnosis and the use of the dark ground microscope. Remember it is the pre-Wassermann stage which alone gives a hundred percentage of success.

### DISEASES OF CHILDREN.

A valuable course of instruction on the above subject, open to post-graduates and to students of the hospital, will be commenced at the London Hospital Medical College on Saturday, January 10, 1920. The teaching will be conducted by Robert Hutchison, M.D., F.R.C.P., Theodore Thompson, M.D., F.R.C.P., and Charles Miller, C.B.E., M.D., F.R.C.P. Dr. Robert Hutchison will give a course of ten lectures on general diseases on Saturdays at 10.15 a.m.; Dr. Thompson a course of eight lectures on organic and functional nervous diseases, meningitis, and mental deficiency, on Mondays at 9.15 a.m.; and clinical demonstrations will be given by Dr. Miller on Wednesdays at 10 a.m.

### THE SECONDARY EFFECTS OF WAR ORGANISATIONS.

We said as soon as the war ended that the future welfare of the voluntary hospitals depended on the survival of the auxiliary aids created by the war. Many of these lapsed, but the centre has remained, and the British Red Cross happily is now married to the voluntary system. This has encouraged and will further encourage local effort, and happy signs of that are general, even in Ireland. For the Irish War Hospital's Supply Depot in Mount Street is to be permanent. This has led to an appeal for additional workers, and as this was recently made in set form and under Sir John Ross's leadership it is likely to be successful. The matrons of the Dublin hospitals have been active in support, and Miss Reeves, matron of Steevens' Hospital, Miss O'Flynn, matron of the Children's Hospital, and Miss Michie, lady superintendent of the Jubilee Nurses, have made a series of useful suggestions. Indirectly, as well as directly, the permanence of the depot will do good. A significant fact at the recent meeting when the future policy was determined was the variety of suggestions made with the object of preserving the voluntary system. One was the case for more district nurses, another the case for amalgamation. In short, active interest in the voluntary principle is the order of the day, and once war enterprises are enlisted there is good hope that each deficiency will be tackled in turn, and that new buttresses will spring to support the structure which the hospitals have built in our national life.

### HOSPITAL SALES.

Among the new developments which should not be allowed to die has been the interest given during the war to sales on behalf of hospitals or the Red Cross. In the old days the goods offered were indeed a jumble, and people were invited to buy the things which nobody wanted. During the war all this was changed. The test came to be the connoisseur's, and the question was whether this or that article was worth admission to the hospital sale-room. There is no reason why this standard should not be preserved. A precedent has been created, and the secretary with intelligence will use that precedent to maintain the interest of any sale which his committee may sanction. It is simply another instance of the value of applying the hospital standard to everything, and of making full use of an improvement which arose during the war. Gifts in kind need not be limited to linen or food or vegetables. Those who cannot subscribe may yet be reminded that their artistic or historic possessions can be worthily offered, and suitably sold in a sale-room which has an object more dignified than the profit of the auctioneer. We shall hope to see the new standard maintained as a matter of course in the future.

### LABOUR'S DILEMMA AT NEWCASTLE.

The working men of Newcastle have long mistaken interference for management, and favoured the creation of numerically inflated committees at which the temptation of members to debate rather than to decide has often been irresistible. Their latest effort has been to seek to control their own house committee by so altering the rule which empowers the house committee to fix the salaries of the lay staff as to give power to "any court of governors to confirm or readjust such salaries." This attempt to interfere with their own appointed executive naturally led to the resignation of the chairman and vice-chairman of the house committee and two other members. The medical staff have fully realised the gravity of the situation, and abortive conferences between doctors, working-men governors and the house committee have been held. Just before Christmas a special court was held. But the principle is so clear that there can be only one decision. The house committee must be left free while it is in office, and it is open to working-men governors who do not approve of its policy not to re-elect the members. Democracy is only possible when the executive is subordinate to its constituents in the long run, but, in the intervals of its office, is left free to take the decisions which are its only reason for existence.

### HOW TO IMPROVE HOUSE-TO-HOUSE COLLECTIONS.

There has been one permanent lesson from the street collections of recent years which should not be lost sight of, namely, the advantage of asking for a definite sum, however small. We believe that this principle would assist the success of the house-to-house collections which certain hospitals organise each year. It is estimated, say, that sixpence per household would raise the required sum of £5,000 in a particular district. If this is so, not only should every house be visited, which is rarely accomplished, but every house should be asked for its particular sixpence, a sum which few would be likely to refuse. In fact, the smallness of the sum would be the chief factor in the success of the collection, which the smaller hospitals might use, in this way, to much greater advantage than they do at present. In place of the somewhat artificial rivalry of two sides of a street, the collector's argument would be that no one had refused, and that if each house would give this definite fraction the money could be raised without worry to the public or unnecessary anxiety to the hospital. The plan at all events is worth trying. Is there any reason to doubt its success?

### A CURIOUS CASE.

In one of the periodic complaints against hospitals which are too full to admit an emergency and have to arrange for the transfer of the patient to another hospital, we noticed a peculiar point. The facts that the patient was dressed on arrival and made comfortable during his brief stay were not disputed; no charge of neglect was substantiated; nor, when the Labour cry that an emergency had been turned away "as if he were a dog," had subsided, was the hospital's account disputed. Why, then, was so much sympathy aroused? The only unusual feature of the case was that it was one of attempted suicide, and, since everything possible was done and the treatment, we believe, successful, we may be permitted to smile at the excessive feeling aroused. It would appear, however, that the Labour Party was on the pounce for some excuse to allege neglect, and was unfortunately abetted by the practitioner who sent the case without previously inquiring whether there was a vacancy. It is odd that the hospital did not publish the correspondence, but left its own indication to the local newspaper, the enterprise of which gave publicity to the letters.

### CLOSING OF A DISPENSARY.

The Wandsworth Common Dispensary, in connection with the Bolingbroke Hospital, which has been in existence for forty years, will be closed at the end of the year. This is due to the falling off in the number of patients since the introduction of the National Health Insurance scheme. From 1,171 in 1909 they have fallen to 587 in 1918, with a further decrease this year. The Secretary announces that the majority of the medical officers are unable to continue their services, which is a further reason for the closing of the dispensary.

### HOSPITAL PHARMACISTS.

A recently-formed body of hospital officials is the Pharmacy Unit of the Incorporated Association of Hospital Officers. The object of the unit is to safeguard the interests of the qualified pharmacists who are employed in the dispensaries of public hospitals. At the first meeting of the session held at 28 Bedford Square, W.C., on November 28, Mr. T. Whitmore Peck, pharmacist to the General Hospital, Birmingham, opened a discussion on the subject of "Salaries of Hospital Dispensers." Representatives of most of the London hospitals were present, as well as many private pharmacists. In stating that at the present time the salaries paid to pharmacist-dispensers are inadequate, Mr. Peck was voicing the general opinion of those present. It is a fact that hospitals are not obliged to appoint pharmacists as dispensers, and can if they choose employ less highly qualified officials. This tends to keep salaries at a lower level. The high cost of living and the reduced purchasing power of money, render a general increase in dispensers' salaries desirable. The January meeting of the Association will be devoted to a discussion on "National Insurance Dispensing."

### POSTS REFUSED FOR LACK OF HOUSES.

Numbers of local bodies are at their wits' end to get doctors. Swansea finds itself in the category. "I am sick and tired of the difficulty," said Alderman Gwynn at a meeting of the Schools Medical Committee. "What is wrong that we cannot get doctors?" The alderman's outburst arose from the fact that out of three applicants for the post of assistant medical officer, only one—a Liverpool applicant—turned up, and he left the committee in doubt as to acceptance of the position in view of the housing difficulty. If local authorities provided quarters for their medical staff, or inserted a guarantee in their advertisements that suitable residence is available, there would doubtless be a better response from medical men.

### DO MOTHERS WANT LECTURES?

At a meeting of the Hull Corporation Maternity and Child Welfare Committee Dr. Jackson mentioned that she had received a letter from the medical officer of health with regard to the giving of lectures at the clinics. She informed the committee that the medical officers at the clinics found their time fully occupied in giving advice to mothers and the children, and further that a considerable amount of time was put in by the medical officers outside the recognised hours of the clinics. It was therefore practically impossible to spend time in giving lectures, and she expressed considerable doubt as to the utility of them, as in the circumstances which prevail it is extremely difficult to get the mothers to stay. If, however, it was an absolute requirement of the Ministry of Health that the lectures should be given, she suggested that they might be given by a competent nurse. The committee decided to ask the medical officer of health to report as to the absolute necessity for arranging lectures to mothers attending the clinics, and whether the time of the medical officers at the clinics is not better spent in giving personal advice to the mother.

### A HOSPITAL PORTER AS COLLECTOR

The Merit Jewel of the M.U.I.O.O.F. has been presented to Bro. John Moss, the head porter of the East Suffolk and Ipswich Hospital, the most successful of the collectors on the Ipswich Friendly Societies' Hospital Sunday. Mr. Moss has been for twenty-one years in hospital work, and has spent nineteen of them at Ipswich.

### THIS WEEK'S DRUG MARKET.

The market has not yet fully recovered from the effects of the holiday, although a fair amount of pressing business has been transacted. When stocktaking is over buying and selling will recommence in earnest, and so far as one can foresee prices are likely to be maintained until well into the New Year at any rate. At the moment a few articles have an easier price tendency, but this is due to temporary absence of demand, and prices are likely to firm up again as soon as the demand sets in. Sandal-wood oil is dearer, consequent upon the rise in the Indian exchange. Orris root is also dearer. Camphor and menthol are for the time being quiet and values are barely maintained.

